# Plane-Stress Deformation Behavior of CoCrFeMnNi High-Entropy Alloy Sheet under Low Temperatures

**DOI:** 10.3390/ma17102259

**Published:** 2024-05-10

**Authors:** Haitao Qu, Yujie Han, Jiaai Shi, Mengmeng Li, Jiayu Liang, Jinghua Zheng

**Affiliations:** 1 AVIC Manufacturing Technology Institute, Beijing 100024, China; 15210565628@163.com (H.Q.); hanyujiehao@126.com (Y.H.); 2 School of Mechanical Engineering, Dalian University of Technology, Dalian 116024, China; homura_zero@126.com (J.S.); lmm722809@163.com (M.L.); 15128205601@163.com (J.L.); 3 College of Material Science and Engineering, Nanjing University of Aeronautics and Astronautics, Nanjing 211106, China

**Keywords:** HEA, bulging, plane stress, deformation, grain

## Abstract

High-entropy alloys are promising candidates expected to be applied in transportation equipment serving in extreme environments due to their excellent properties. CoCrFeMnNi high-entropy alloy is a typical representative of them, and its low temperature performance is excellent. In this study, to evaluate the feasibility of forming HEA shells, the deformation behavior of CoCrFeMnNi under a plane-stress state at lower temperatures was thoroughly studied. Firstly, a thin-walled HEA tube was fabricated using hot extrusion and further formed into a thin shell for uniaxial tensile and biaxial bulging tests. Subsequently, uniaxial tensile tests at cryogenic temperatures were conducted. Both the strength and the ductility improves as the temperature decreases from −160 °C to −196 °C. Then, a systematic low-temperature bulging test was performed using isothermal dome tests and the thickness uniformity analysis of the bulged specimens was carried out. In addition, grain microstructural observation using EBSD was characterized analyze the possible deformation mechanism at the cryogenic temperature under the biaxial stress state. This study, for the first time, investigated the biaxial deformation behavior of HEA. Considering the plane-stress state deformation is the dominant type in the thin-walled shell deformation, this study enables us to provide direct guidance for various sheet-forming processes of HEAs.

## 1. Introduction

High-entropy alloys (HEAs) are newly developed advanced materials [1]. Differing from the classical alloys whose number of base elements is only one or, rarely two, and HEAs have several kinds of principal elements [2]. HEAs have many outstanding properties, such as high strength, high hardness, excellent corrosion resistance, excellent fatigue and so on [3]. In the high- or cryogenic-temperature environment, HEAs show excellent mechanical properties as well [2,4], which brings great HEA manufacturing potential in terms of structural components in various industrial sectors [2].

Thin-walled shell materials, e.g., tubes and sheets, are important structural types of raw materials, which can be further manufactured into various industrial products. Cold-rolling and annealing are significant methods to fabricate HEA sheets and improve the mechanical properties as well. Recently, there have been several investigations about the fabrication process of HEA sheets. Klimova et al. [5] found that the yield strength, the ultimate strength and the fracture elongation of as-casted Al and C-Containing CoCrFeNiMn-type HEAs are 210 MPa, 455 MPa and 80%, respectively, and after rolling with the reduction of 80%, the yield strength and the ultimate strength increase to 1310 MPa and 1500 Mpa, respectively, while the fracture elongation decreases to 6.5%. Shabani et al. [6] found that the ultimate tensile strength of the as-homogenized FeCrCuMnNi HEA is approximately 500 MPa, and when it is cold-rolled with the deformation of 85%, the ultimate strength increases by approximately 160%. Zou et al. [7] found that after cold-rolling and post-deformation annealing, for the casted CrMnFeCoNi, which is homogenized and quenched, the yield strength increases from 209 MPa to 352 MPa and the ultimate tensile strength increases from 566 MPa to 702 MPa, while the elongation decreases from 59.3% to 43.8%. Wu et al. [8] respectively cryorolled and cold-rolled as-casted CoCrFeNiMn HEA to prepare sheets and found that the yield strength increased from 161 MPa to 1259 MPa and 1082 MPa, respectively, and after annealing at 873 K, both the yield strength and the ultimate tensile strength of the cryorolled HEA were greater than those of the cold-rolled HEAs.

Among various superior properties, significantly enhanced mechanical properties at cryogenic temperatures are the most attractive. Understanding the mechanical properties of HEAs at cryogenic temperature is also helpful for the employment of HEAs in cryogenic forming [4]. Gali et al. [9] found that when the engineering strain rate is 10^−3^/s, the tensile ductility of both CrMnFeCoNi and CrFeCoNi decreases from higher than 60% to lower than 35% as the temperature increases from 77 K to 1273 K. Otto et al. [10] found that the strength of the CoCrFeMnNi HEA has strong temperature dependence when the temperature is in the range from 77 K to 1073 K, and when the temperature is 77 K, the 0.2% offset yield strength, the ultimate tensile strength and the fracture elongation all reach the maximum. Laktinova et al. [11] found that when the temperature decreases from 300 K to 4.2 K, the nominal yield strength of the Ag0.5 CoCrCuFeNi HEA increases from 450 MPa to 750 MPa. Gludovatz et al. [12] found that as the temperature decreases from 293 K to 77 K, the yield strength, the ultimate tensile strength and the tensile ductility of the CrMnFeCoNi HEA increase approximately 85%, approximately 70% and approximately 25%, respectively, and when the temperature is 77 K, the yield strength and the ultimate tensile strength of the CrMnFeCoNi HEA are 759 MPa and 1280 MPa, respectively. Laplanche et al. [13] found that as the temperature decreases from 293 K to 77 K, the engineering yield strength of the CrMnFeCoNi HEA increases from 265 ± 10 MPa to 460 ± 30 MPa, the ultimate tensile strength increases from 600 ± 40 MPa to 1060 ± 70 MPa, and the tensile ductility increases by approximately 50% as well. The above investigations show that researches about the mechanical properties of the HEAs at cryogenic temperatures at the uniaxial stress state has been relatively consummate, and the HEAs show a relatively high strength and ductility elongation, i.e., relatively good formability at cryogenic temperatures.

However, most of the above investigations on the mechanical properties of HEAs focused on the uniaxial tensile stress state at cryogenic temperatures, while few investigations of HEAs about the biaxial tensile stress state were identified [14], which is more familiar in the forming process of sheet metals. In this study, the deformation behavior of CoCrFeMnNi HEA in the plane stress state at the cryogenic temperature is investigated. Firstly, the uniaxial tensile tests of the CoCrFeMnNi HEA were carried out at the cryogenic temperature, whose specimens were cut from the fabricated thin-walled tubes. Then, the bulging tests at the cryogenic temperature for the CoCrFeMnNi HEA were carried out, and the effect of various parameters on the thickness uniformity was investigated. Finally, the grain microstructural observation using EBSD was carried out to analyze the possible deformation mechanism at the cryogenic temperature under the biaxial stress state. This study firstly investigated the biaxial deformation behavior of HEA, which is utilized in the process of shell deformation, and can provide direct theoretical guidance for the entire forming processes at the cryogenic temperature of HEA shells, which may be employed in aerospace applications with extreme environments in the future.

## 2. Materials and Methods

### 2.1. Material

**Figure 1:**
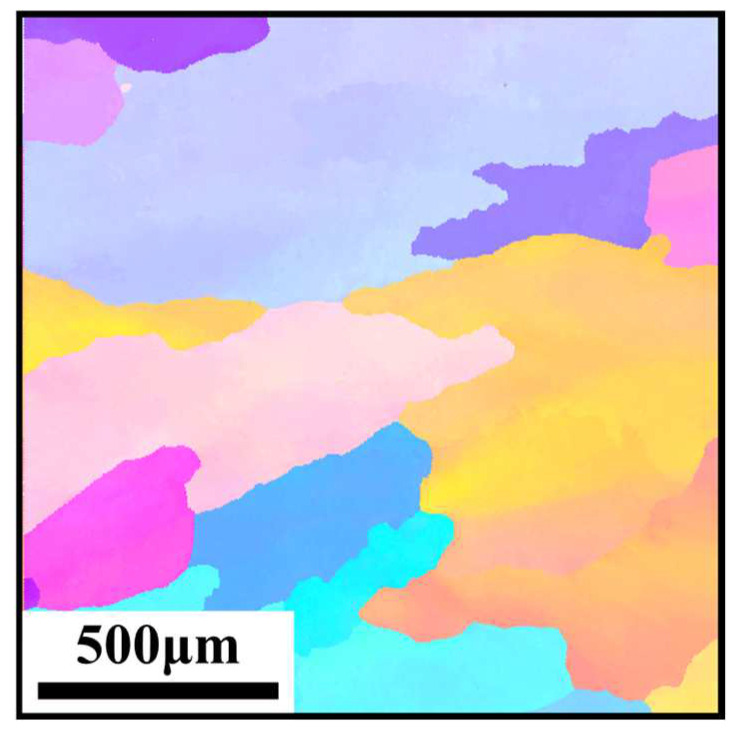
The microstructure of the as-casted CoCrFeMnNi HEA.

The basic CoCrFeMnNi five-element high-entropy alloy used in this study was prepared by vacuum induction arc melting in Shenzhen Sematic New Material Company (Shenzhen, Guandong, China). The chemical composition and the corresponding mass fractions of the CoCrFeMnNi HEA are shown in Table 1, and the microstructure of the as-casted HEA is shown in Figure 1. Firstly, a cylindrical billet with an outer diameter of 39.2 mm considering thermal expansion, an inner diameter of 25 mm and a height of 50 mm was machined by wire cutting on a large ingot. The samples were subjected to vacuum homogenization annealing heat treatment in the condition of 1200 °C × 24 h, to remove the casting defects and optimize the microstructure.

### 2.2. Fabrication of HEA Hot Extrusion Tube

**Figure 2:**
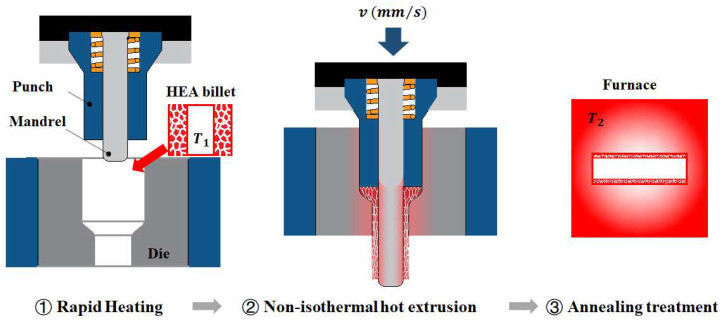
The manufacturing process of the HEA extrusion thin-walled tube.

Thin-walled tubes were used as the investigated raw material type in this study, which was fabricated using hot extrusion process, as schematically shown in Figure 2. The hot extrusion process was carried out in Dalian University of Technology (Dalian, Liaoning, China). During the hot extrusion process, the HEA billet was firstly heated in the furnace with the preset temperature of 950 °C and then soaked for half an hour. Then, the billet was quickly transferred from the heating furnace to the extrusion die. The position of the punch should be adjusted properly to avoid the heat loss of the billet before hot extrusion. The billet was extruded and deformed to the tube by extrusion dies. The extrusion speed was 15 mm/s and the extrusion ratio was 3.01. Finally, to remove the extruded elongated microstructure and reduce the work hardening, annealing treatment in the condition of 950 °C × 1 h was given for all the extruded tubes.

**Figure 3:**
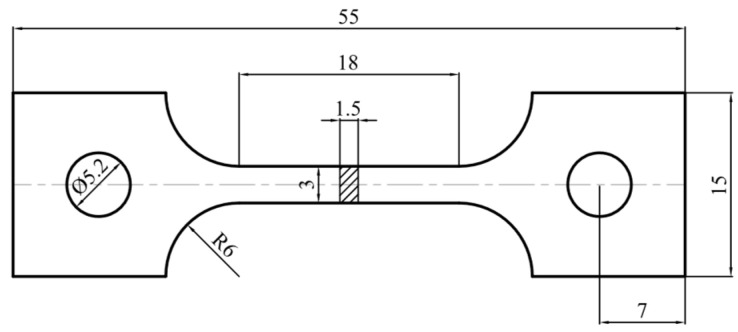
The shape and dimensions of the cryogenic uniaxial tensile specimen cut from the HEA hot extrusion tube (unit: mm).

To evaluate the mechanical properties at cryogenic temperatures, the uniaxial tensile tests were designed and were carried out in Technical Institute of Physics and Chemistry, CAS (Beijing, China). The uniaxial tensile test specimens were directly cut from the extruded tubes and the shape and the dimensions of the specimen are shown in Figure 3. The cryogenic uniaxial tensile tests were carried out at the temperatures of −160 °C and −196 °C, which is the lowest achievable deformation temperature under the liquid nitrogen environment in the low-temperature box.

### 2.3. Cryogenic Bulging Tests

#### 2.3.1. Design and Preparation of Bulged Specimen

To machine circular specimen for bulging tests, circular sheets were firstly made from the extruded tubes. The tubes were cut into 4 pieces by wire cutting, and then the cut tubes were flattened at room temperature by press. Finally, the sheets were machined to specific size by wire cutting. According to the size of the HEA thin-walled tubes, the diameter of the plate was determined to be 42 mm, the thicknesses of the plates were measured, and the initial average thicknesses were obtained.

Before testing, the surfaces of the specimens were cleaned to be free of impurities and smooth. Then, the appropriate point etching solution was selected, and a uniform origin grid with the diameter of 1 mm was printed on the specimen surface by electric etching. In the grid-painting process, ARGUS grid printing was used.

#### 2.3.2. Test Set-Up and Program of Cryogenic Bulging

**Figure 4:**
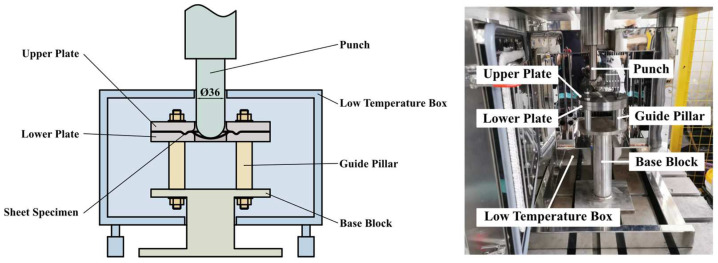
The schematic diagram and the substance of the experimental equipment of the bulging tests at cryogenic temperatures.

Figure 4 shows the schematic diagram and the substance of the experimental equipment of the bulging tests at cryogenic temperatures, which is in Dalian University of Technology (Dalian, Liaoning, China). The bulging set-up is mainly composed of the punch, the upper plate, the lower plate, the guide pillar and the base block. To achieve the plane-stress bulging deformation, the sheet specimen is firmly com-pressed by the draw beads designed on the upper and lower plates. To provide sufficient clamping force, the two plates are initially clamped using the press force and then fixed by four bolts. This assembly is mounted on the base box, which is embedded in the cryogenic environment. The minimum achievable environment temperature is −190 °C, and the temperature accuracy is 1 °C. Once the sheet is firmly clamped, the central spherical punch with the diameter of 36 mm is used to bulge the specimen. The punch is fixed directly to the press, which is able to program the stroke and forming speed.

In this study, both room-temperature and cryogenic-temperature bulging tests were performed. Three temperatures, −190 °C, −140 °C and RT (room temperature), were selected. Two kinds of tests were designed. First, the specimens were bulged to splitting at different temperatures. During bulging, the force and the stroke were recorded. Then, to obtain microstructure at various bugling deformation extensions, interrupted bulging tests stopped at specific strokes under various temperatures were carried out.

### 2.4. Sampling Scheme of Post-Processing

**Figure 5:**
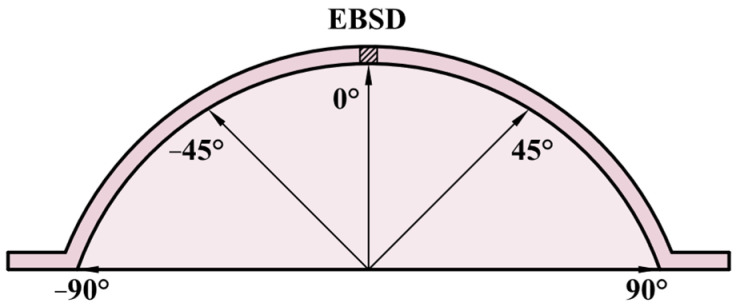
Sampling scheme for thickness measurement and EBSD observation.

The thickness distribution of the bulged specimen is an important index to evaluate the deformation uniformity, so it is necessary to measure the thickness of the bulged specimens. The microstructure is also significantly affected by the bulging deformation, so the EBSD observation was carried out as well. Figure 5 shows the sampling scheme for thickness measurement and EBSD observation. The bulged specimen was cut in the middle, and then the point measurement scheme was given on the bulged dome every 5°. The thickness data of each point were measured and compared for different bulging conditions. The area near 0° was chosen to carry out the EBSD observation.

## 3. Results and Discussion

### 3.1. Uniaxial Tensile Tests

**Figure 6:**
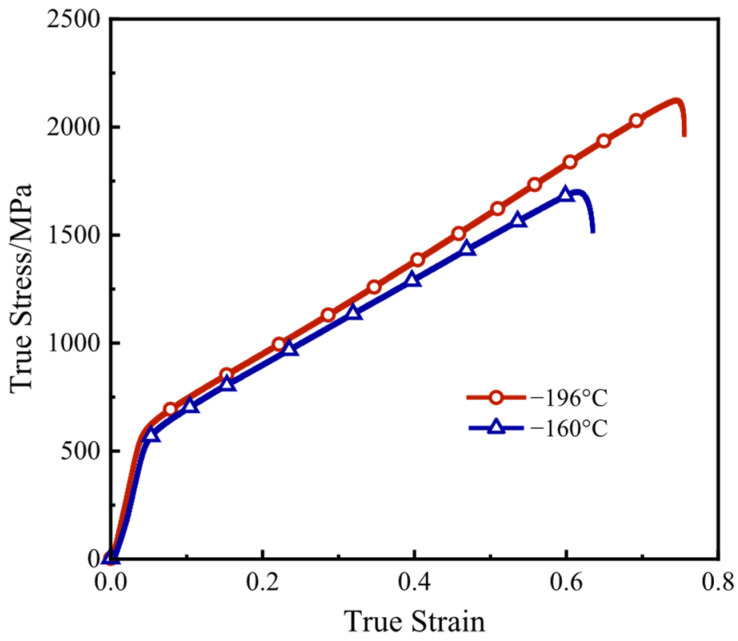
Low-temperature uniaxial tensile stress–strain curves at different temperatures.

Figure 6 shows the low-temperature tensile test results of HEA thin-walled tubes. It can be seen that this material exhibits excellent mechanical properties at low temperatures. The yield strength of the alloy at −160 °C can exceed 500 MPa, the ultimate tensile strength is near 1700 MPa and the elongation is approximately 60%. When the temperature decreases to −196 °C, the ultimate tensile strength can be greater than 2100 MPa, and the elongation can be higher than 70%. For most metallic materials, ductility and strength always have negative correlation, but the obtained tensile test results of the CoCrFeMnNi HEA at cryogenic temperature shows the opposite characteristics, which is similar to previous investigation results of HEA mechanical properties [9,10,12,13,15]. As the temperature decreases from RT to 77 K, the mechanism of the plastic deformation of the HEA changes from dislocation gliding to the mix of dislocation gliding and nanoscale twinning [10]. The appearance of nanoscale twinning may postpone the onset of necking [9,13], which may be the reason for the increase in both the strength and the ductility as the temperature decreases from −160 °C to −196 °C.

### 3.2. Plane-Stress Bulged Domes at Various Low Temperatures

**Figure 7:**
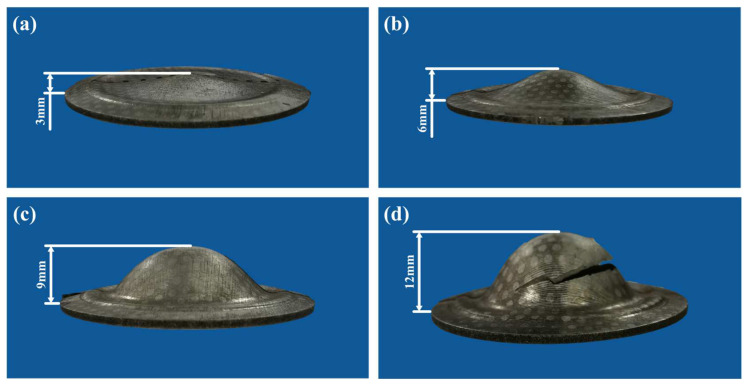
Bulged specimens at the temperature of −190 °C under the bulging ratio of (**a**) 25%, (**b**) 50%, (**c**) 75% and (**d**) 100%.

**Figure 8:**
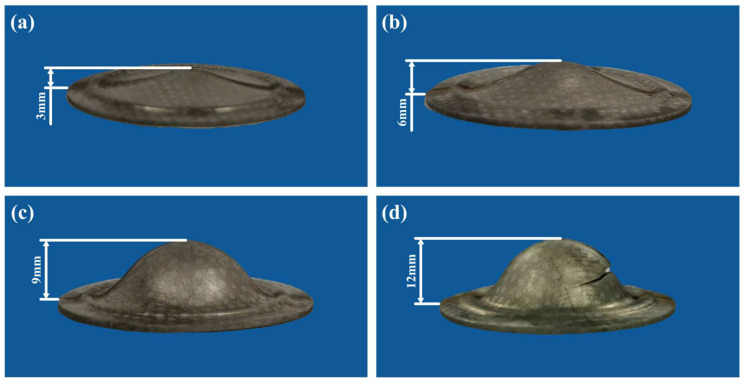
Bulged specimens at the temperature of −140 °C under the bulging ratio of (**a**) 25%, (**b**) 50%, (**c**) 75% and (**d**) 100%.

The process parameters of the bulging tests at the cryogenic temperature are shown in Table 2. Two temperatures of −190 °C and −140 °C were chosen. The ultimate bulging heights once splitting occurs at the two temperatures are both 12 mm. The interrupted bulging tests under various actual bulging heights were also carried out, and the bulging ratio is given to express the proportional relationship between the actual bulging height and the ultimate bulging height. Four kinds of actual bulging heights were chosen, whose corresponding bulging ratios are 25%, 50%, 75% and 100%, respectively. All the experimental results at the temperatures of −190 °C and −140 °C are, respectively, shown in Figure 7 and Figure 8.

### 3.3. Thickness Distributions at Various Low Temperatures

The thinning rate of the bulged specimens R can be calculated using Equation (1), in which the t is the thickness of the bulged specimen at various positions, and the $t_{0}$ is the initial average thickness of the sheet specimen:

(1) $$R=\frac{{\bar{t}}_{0}-t}{{\bar{t}}_{0}}\times 100\%$$

The initial average thicknesses of the sheet specimens used in the bulging tests at the cryogenic temperature under various process parameters are shown in Table 3.

**Figure 9:**
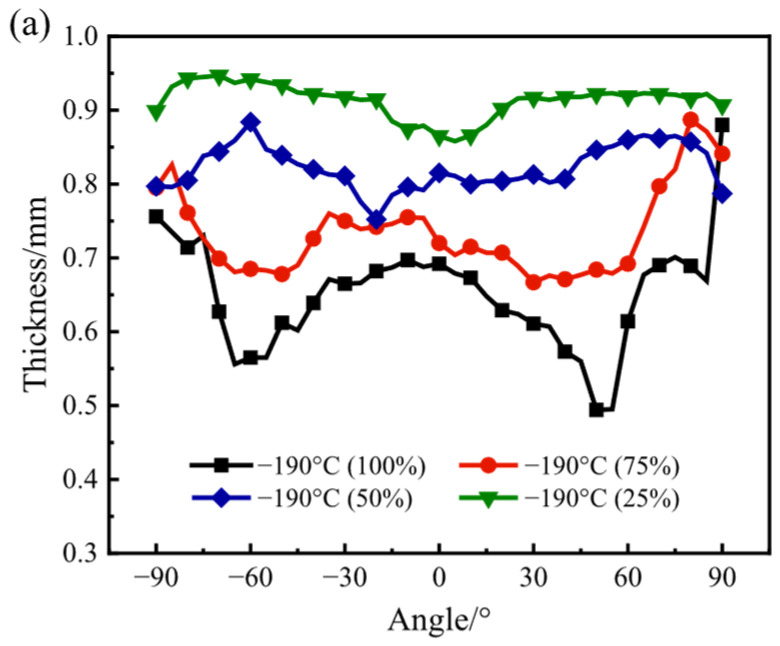

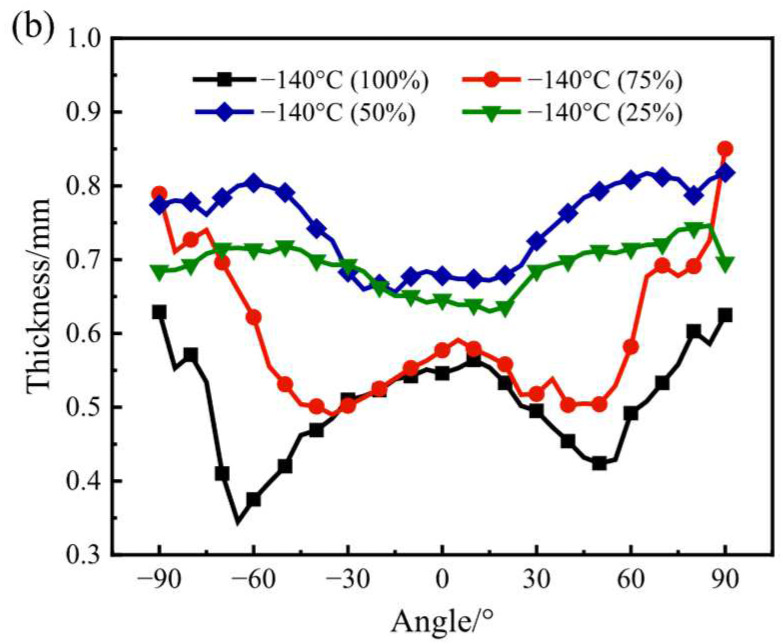
The distribution of the thickness under various bulging ratios at the temperatures of (**a**) −190 °C and (**b**) −140 °C.

Figure 9a and Figure 9b, respectively, show the distribution of the thickness of the bulged specimens under various bulging ratios at the temperatures of −190 °C and −140 °C. The bulged specimens whose bulging ratio is 25% show the best thickness uniformity, at both temperatures of −190 °C and −140 °C, compared with those whose bulging ratios are higher. In addition, both specimens with a bulging ratio of 25% at the temperature of −190 °C and −140 °C show similar distribution laws of the thickness. At the area near 0°, the thickness reaches the minimum. With the increase in the absolute value of the angle, the thickness firstly increases broadly and then slightly decreases. At the area whose angle is ±90°, the value of the thickness reaches the minimum. When the temperature is −190 °C, with the increase in the bulging ratio, the thickness of the bulged specimen decreases broadly. However, when the temperature is −140 °C, as the bulging ratio increases from 25% to 50%, the value of the thickness slightly increases, and then as the bulging ratio increases from 50% to 100%, the value of the thickness broadly decreases. When the bulging ratio reaches 75% and even 100%, compared with the lower bulging ratios, there is a significant variation in the distribution of the thickness. The minimum thickness appears at the area near ±60°, and there is a maximum at the area near 0°. At the area near ±90°, the value of the thickness reaches the maximum. The reason for the variation of the distribution of the thickness with the increase in the bulging ratio is the variation of the friction between the area near 0° and the punch. When the bulging ratio is relatively small, the relatively small deformation of the bulging specimen firstly occurs at the area near 0°. The pressure between the area near 0° and the punch is relatively small, and the friction is relatively small as well. As the bulging ratio increases, the deformation of the bulged specimen becomes larger. The pressure between the area near 0° and the punch becomes larger, and the friction becomes larger as well. As a result, the deformation of the area near 0° becomes smaller than that near 60°, which is less affected by the friction from the punch.

**Figure 10:**
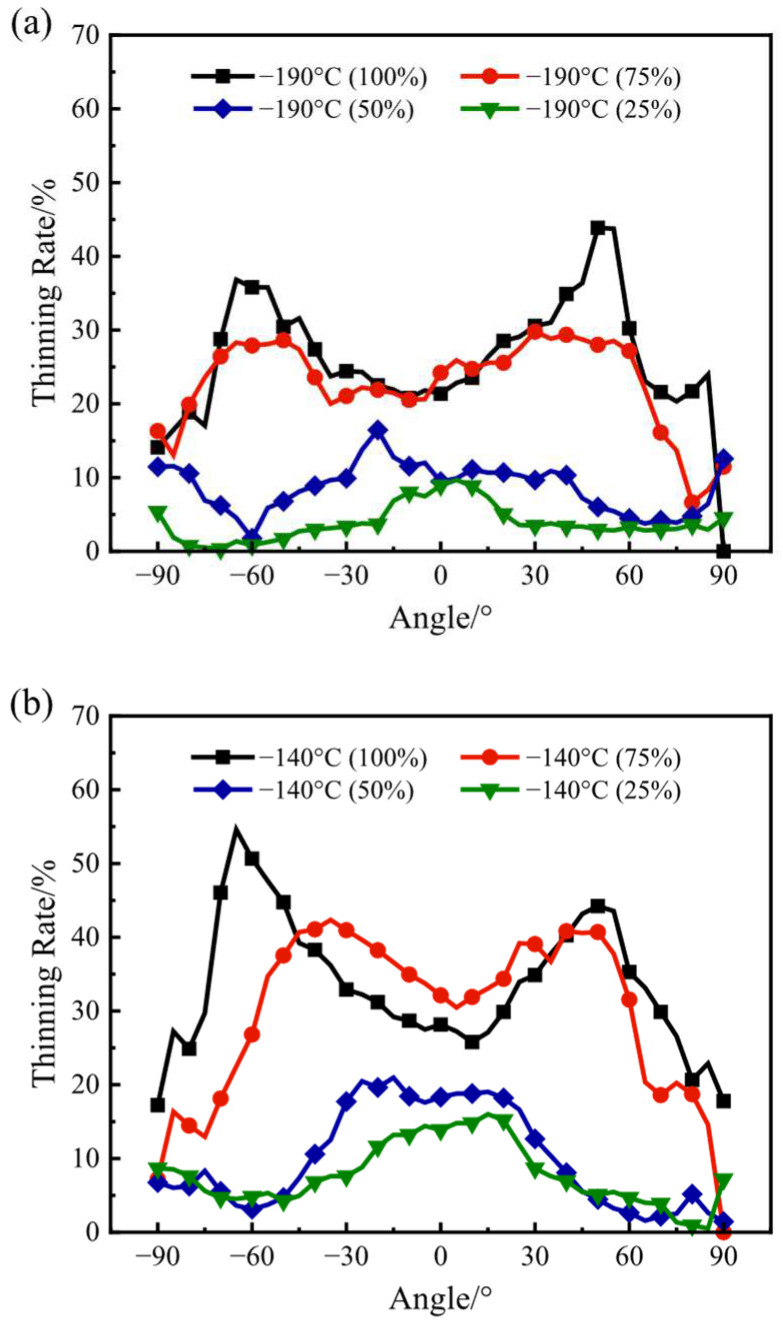
The distribution of the thinning rate under various bulging ratios at the temperatures of (**a**) −190 °C and (**b**) −140 °C.

To further investigate the effect of the bulging ratio on the distribution of the thickness, the thinning rate of the thickness at various areas under various bulging ratios at the temperatures of −190 °C and −140 °C was calculated with the equation and the measured initial average thickness, and the results are shown in Figure 10a,b. When the temperature is the same, with the increase in the bulging ratio, the deformation extent of the bulged specimen increases, and the thinning rate broadly increases as well. On the premise of the same bulging ratio, the thinning rate of the bulged specimen whose bulging temperature is −190 °C is broadly lower than that whose bulging temperature is −140 °C. At the temperature of −190 °C, the maxima of the thinning rate are, respectively, 6.32%, 4.52%, 12.56% and 10.74% smaller than that at the temperature of −140 °C at the bulging ratios of 25%, 50%, 75% and 100%, respectively. It can be obtained that the uniformity of the thickness of the bulged specimen whose bulging temperature is −190 °C is broadly better than that whose bulging temperature is −140 °C. Furthermore, both the strength and the plasticity of the HEA broadly increase as the temperature decreases from −140 °C to −190 °C in the biaxial tensile stress state, which is similar to that in the uniaxial tensile stress state. When the bulging ratio is 25% and 50%, which is relatively low, the thinning rate reaches the maximum at the area near 0°. The reason for this is that the middle of the sheet specimen firstly deforms and transitions to the biaxial tensile stress state when the bulging ratio is relatively low. With the increase in the absolute value of the angle, the thinning rate firstly decreases and then slightly increases, and at the area near ±90°, the value of the thickness reaches the maximum. When the bulging ratio is 75% and 100%, which is relatively high, the distribution of the thinning rate significantly changes. At the area near 0°, the value of the thinning rate reaches the minimum. As the absolute value of the angle increases to approximately 60°, the value of the thinning rate reaches the maximum. The reason for this is that when the bulging ratio is relatively higher, the areas whose absolute value of the angle is 0° and 60° are both in the biaxial tensile stress state; in addition, compared with the area near 0°, the area near ±60° is less affected by the friction between the specimen and the punch. As the absolute value of the angle increases from 60° to 90°, the value of the thinning rate decreases broadly. The reason for this is that the area whose absolute value of the angle is approximately 90° is near the edge of the bulged specimen, which is more affected by both the upper and the lower plate and is in the tensile-compression-stress state instead of biaxial tensile stress state.

**Figure 11:**
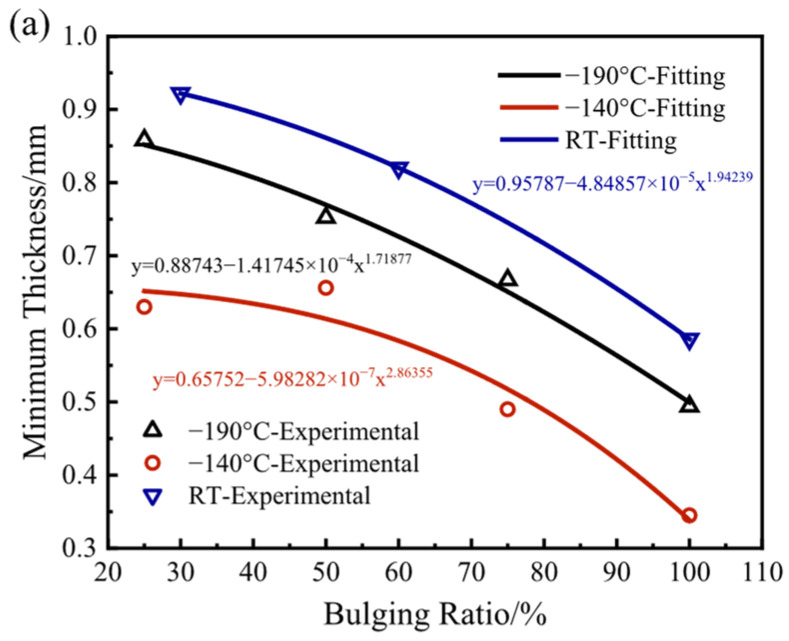

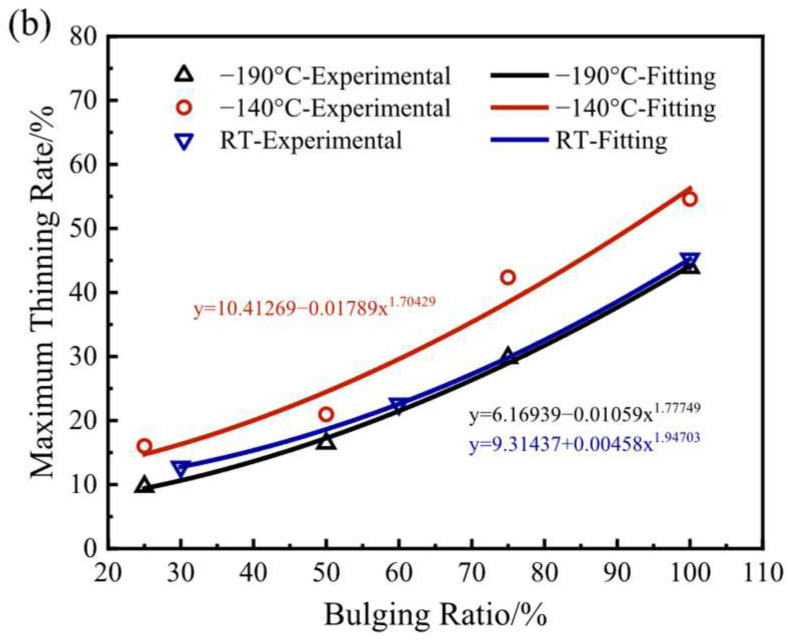
The comparisons between the fitting calculation value and the experimental results of the (**a**) minimum thickness and the (**b**) maximum thinning rate.

Figure 11a,b show the comparisons between the fitting calculation values and the experimental results of the minimum thickness and the maximum thinning rate under various bulging ratios and temperatures. The fitting equations under various bulging ratios and temperatures are also given in the figures, whose forms are all y = A + Bx^C^. When the bulging temperature is the same, with the increase in the bulging ratio, the minimum thickness decreases, and the maximum thinning rate increases because of larger deformation. When the bulging temperature is −140 °C, the minimum thickness is lower, and the maximum thinning rate is higher than those at other temperatures. When the bulging temperature is −190 °C, the maximum thinning rate is broadly lower than those at other temperatures because of higher strength and plasticity. The experimental results are in good agreement with the fitting calculation value, which verifies the reliability of the fitting curve.

### 3.4. EBSD Microstructure Analysis

**Figure 12:**
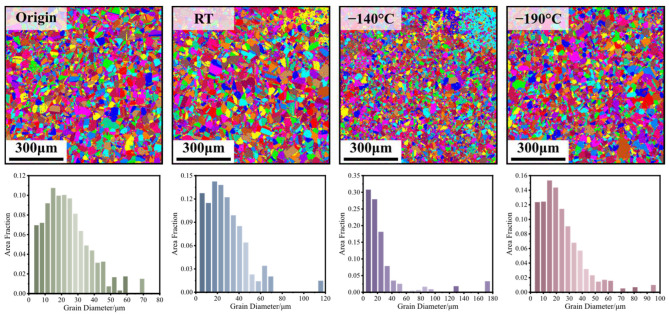
The EBSD observations and the probability density distribution of the grain diameters under the original state and the working conditions with various bulging temperatures.

**Figure 13:**
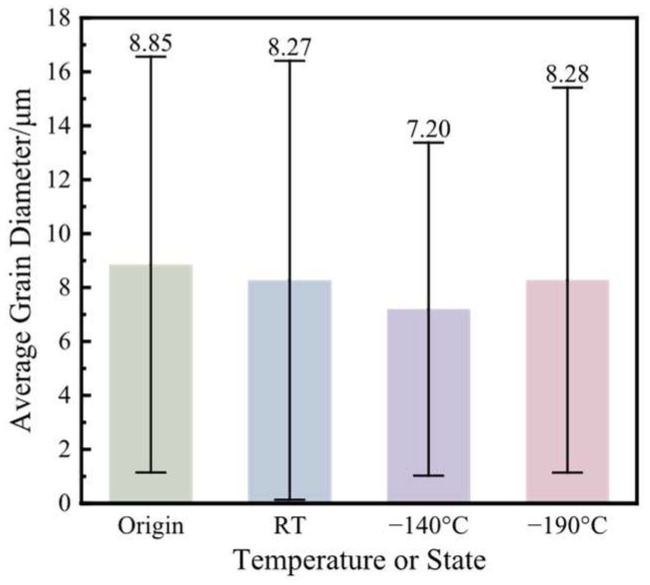
The statistical results of the average grain diameter and the standard deviation under the original state and various bulging ratios.

Figure 12 shows the EBSD observations and the probability density distribution of the grain diameters at the area near 0° under the original state and the working conditions with various bulging temperatures, and the statistical results of the average grain diameter and the standard deviation are shown in Figure 13. The shapes of the grains are all approximately equiaxed in the original state and the working conditions with various bulging temperatures. In the original state, compared with other bulged working conditions, the average grain diameter reaches the largest value, 8.85 μm. In addition, the grain diameter is not uniform, whose highest and lowest deviations are extremely large. The minimum grain diameter can reach smaller than 10 μm, while the maximum grain diameter can be larger than 60 μm, and the standard deviation of the grain diameter is 7.71 μm. In general, after being deformed, the grains are broken, and the dimensions of the grains become obviously smaller. However, in these obtained results, no matter whether at RT or the cryogenic temperature, the average grain diameter only decreases minimally compared with that in the original state. Furthermore, the grain diameters at each bulging temperature are not uniform. When the bulging temperature is RT, the average grain diameter is 8.27 μm. The minimum grain diameter can be smaller than 10 μm, while the maximum grain diameter can be larger than 110 μm, and the standard deviation of the grain diameter is 8.14 μm. When the bulging temperature is −140 °C, the average grain diameter is 7.20 μm. The minimum grain diameter can be smaller than 10 μm, while the maximum grain diameter can be larger than 170 μm, and the standard deviation of the grain diameter is 6.17 μm. When the bulging temperature is −190 °C, the average grain diameter is 8.28 μm. The minimum grain diameter can be smaller than 10 μm, while the maximum grain diameter can be larger than 90 μm, and the standard deviation is 7.14 μm. It can be obtained that the difference in the grain diameters among the original state and the working conditions at various bulging temperatures is not obvious. Furthermore, the variation in the shape and the dimensions of the grains may not be the main mechanisms of the plasticity deformation, and the grain size may not be the main factor affecting the mechanical properties of the CoCrFeMnNi HEA.

**Figure 14:**
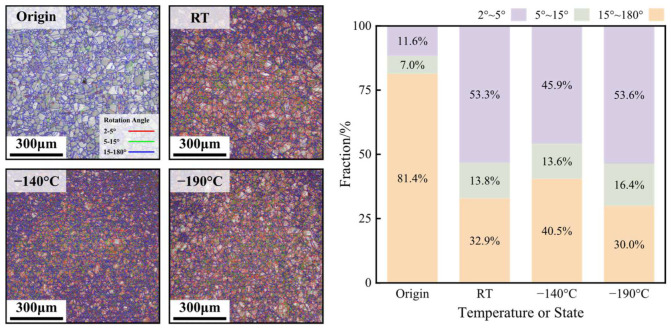
The EBSD grain boundary maps and statistical results of the ratio of the grain boundaries with various angles under the original state and the working conditions with various bulging temperatures.

Figure 14 shows the EBSD grain boundary maps and the statistical results of the ratio of the grain boundaries with various angles at the area near 0° under the original state and the working conditions with various bulging temperatures with the bulging ratio of 100%. In the original state, since the HEA has been annealed, most of the grain boundaries are large-angle grain boundaries whose angle is in the range of 15°~180°, and its ratio can reach 81.4%; the ratio of the small-angle grain boundaries whose angle is in the range of 2°~5° is only 11.6%. After bulging deformation, the ratio of the small-angle grain boundaries significantly increases. Commonly, small-angle grain boundaries can be considered to be composed of a series of edge dislocations, screw dislocations or the net of mixed dislocation [16]. Therefore, it can be obtained that there are a number of dislocations significantly accruing and accumulating, and the dislocation motion may be one of the main mechanisms of the biaxial deformation of the CoCrFeMnNi HEA. However, with the decrease in the bulging temperature, the ratio of the small-angle grain boundaries has no obvious variation. When the bulging temperatures are RT, −140 °C and 190 °C, the ratios of the small-angle grain boundaries are 53.3%, 45.9% and 53.6%, respectively. It may be obtained that as the temperature decreases from RT to −196 °C, the effect of the dislocation motion of the biaxial deformation is not weakened, while the effect of the nanoscale twinning, which is difficult to be observed with EBSD [13], may be enhanced and may be the cause of the improvement in the plasticity of the CoCrFeMnNi HEA at cryogenic temperatures.

**Figure 15:**
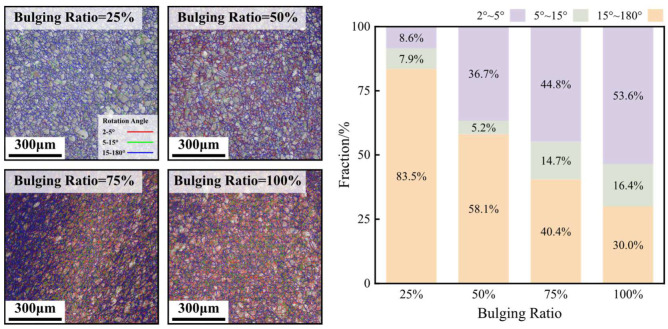
The EBSD grain boundary maps and statistical results of the ratio of the grain boundaries with various angles under various bulging ratios at a temperature of −190 °C.

Figure 15 shows the EBSD grain boundary maps and the statistical results of the ratio of the grain boundaries with various angles at the area near 0° under various bulging ratios at the temperature of −190 °C. It can be obtained that the bulging ratio significantly affects the ratio of the small-angle grain boundaries. As the bulging ratio increases from 25% to 100%, the ratio of the small-angle grain boundaries whose angle is in the range of 2°~5° increases from 8.6% to 53.6%. When the bulging ratio is 25%, the deformation of the HEA is relatively small, there are few dislocations accruing or accumulating and there are few small-angle grain boundaries generating. As the bulging ratio increases, the deformation of the HEA increases, and the dislocations accrue more and accumulate. As a result, there are more small-angle grain boundaries generating, and the ratio of the small-angle grain boundaries significantly increases. In addition, the increase rate of the ratio of the small-angle grain boundaries is not uniform with the increase in the bulging ratio. As the bulging ratio increases from 25% to 50%, the ratio of the small grain boundary increases 28.1%. However, as the bulging ratio increases from 50% to 100%, the ratio of the small grain boundary only increases 16.9%.

## 4. Conclusions

In this study, for the first time, the plane-stress deformation behavior of the CoCrFeMnNi HEA tubes under low temperatures was experimentally investigated using bulging tests. The main conclusions are as follows:

1. For the uniaxial tensile properties at cryogenic temperatures, when the temperature is −160 °C, the yield strength can exceed 500 MPa, the tensile strength is near 1700 MPa and the elongation is approximately 60%. When the temperature decreases to −196 °C, the tensile strength can be greater than 2100 MPa, and the elongation can be higher than 70%. The appearance of nanoscale twinning may postpone the onset of necking, which may be the reason for the increases in both the strength and the ductility as the temperature decreases.
2. For the plane-stress bulging behavior at cryogenic temperatures, the thinning rate at the temperature of −190 °C is broadly smaller than that at the temperature of −140 °C, which means that the thickness is more uniform, and the strength and the plasticity also increase in the biaxial stress state as the temperature decreases from −140 °C to −190 °C. When the bulging ratio is 25% and 50%, because the middle of the specimen firstly deforms and transitions into the biaxial tensile stress state, the thinning rate reaches the maximum at the area near 0°. When the bulging ratio is 75% and 100%, the areas near 0° and ±60° are both in the biaxial tensile stress state, but the area near ±60° is less affected by the friction between the specimen and the punch, so the thinning rate reaches the maximum at the area near ±60°.
3. For the microstructure, the difference in the grain diameter between the original state and the working conditions with various bulging temperatures is not obvious. As the bulging temperature decreases, the ratio of the small grain boundaries has no obvious variation. When the bulging ratio is 100%, at the temperature of RT, −140 °C and −190 °C, the ratios of the small-angle grain boundaries are 53.3%, 45.9% and 53.6%, respectively. The bulging ratio significantly affects the ratio of the small grain boundaries. With the increase in the bulging ratio, the ratio of the small-angle grain boundaries increases from 8.6% to 53.6%.

## Figures and Tables

**Table 1:** The chemical composition of the CoCrFeMnNi HEA.

Element	Co	Cr	Fe	Mn	Ni
Mass Fraction/%	Bal. (20.73)	18.44	20.06	19.79	20.98

**Table 2:** Process parameters of the bulging tests.

Temperature/°C	Bulging Ratio/% (Actual Bulging Height/mm)
−190/−140	25 (3), 50 (6), 75 (9), 100 (12)

**Table 3:** Initial average thicknesses of the sheet specimens.

Temperature/°C	Bulging Ratio/%	Initial Average Thickness/mm
−190	25	0.95
	50	0.90
	75	0.95
	100	0.88
−140	25	0.75
	50	0.83
	75	0.85
	100	0.76

## Data Availability

Data is contained within the article.

## References

[1] Yeh J.-W. (2013). Alloy Design Strategies and Future Trends in High-Entropy Alloys. JOM.

[2] Ye Y.F., Wang Q., Lu J., Liu C.T., Yang Y. (2016). High-Entropy Alloy: Challenges and Prospects. Mater. Today.

[3] Zhang W., Liaw P.K., Zhang Y. (2018). Science and Technology in High-Entropy Alloys. Sci. China Mater..

[4] Lyu Z., Fan X., Lee C., Wang S.-Y., Feng R., Liaw P.K. (2018). Fundamental Understanding of Mechanical Behavior of High-Entropy Alloys at Low Temperatures: A Review. J. Mater. Res..

[5] Klimova M., Stepanov N., Shaysultanov D., Chernichenko R., Yurchenko N., Sanin V., Zherebtsov S. (2017). Microstructure and Mechanical Properties Evolution of the Al, C-Containing CoCrFeNiMn-Type High-Entropy Alloy during Cold Rolling. Materials.

[6] Shabani A., Toroghinejad M.R., Shafyei A., Cavaliere P. (2018). Effect of Cold-Rolling on Microstructure, Texture and Mechanical Properties of an Equiatomic FeCrCuMnNi High Entropy Alloy. Materialia.

[7] Zou Y., Li S., Liu S., Li J., Li Y. (2021). Improved Mechanical and Corrosion Properties of CrMnFeCoNi High Entropy Alloy with Cold Rolling and Post Deformation Annealing Process. J. Alloys Compd..

[8] Wu Y., Luo K., Zhang Y., Kong C., Yu H. (2022). Microstructures and Mechanical Properties of a CoCrFeNiMn High-Entropy Alloy Obtained by 223 K Cryorolling and Subsequent Annealing. J. Alloys Compd..

[9] Gali A., George E.P. (2013). Tensile Properties of High- and Medium-Entropy Alloys. Intermetallics.

[10] Otto F., Dlouhý A., Somsen C., Bei H., Eggeler G., George E.P. (2013). The Influences of Temperature and Microstructure on the Tensile Properties of a CoCrFeMnNi High-Entropy Alloy. Acta Mater..

[11] Laktionova M.A., Tabchnikova E.D., Tang Z., Liaw P.K. (2013). Mechanical Properties of the High-Entropy Alloy Ag_0.5_CoCrCuFeNi at Temperatures of 4.2–300 K. Low Temp. Phys..

[12] Gludovatz B., Hohenwarter A., Catoor D., Chang E.H., George E.P., Ritchie R.O. (2014). A Fracture-Resistant High-Entropy Alloy for Cryogenic Applications. Science.

[13] Laplanche G., Kostka A., Horst O.M., Eggeler G., George E.P. (2016). Microstructure Evolution and Critical Stress for Twinning in the CrMnFeCoNi High-Entropy Alloy. Acta Mater..

[14] Lu P., Zhang T.W., Zhao D., Ma S.G., Li Q., Wang Z.H. (2020). Mechanical Behaviors and Texture Evolution of CoCrFeNi High-Entropy Alloy under Shear-Tension Deformation. J. Alloys Compd..

[15] Li D., Zhang Y. (2016). The Ultrahigh Charpy Impact Toughness of Forged AlxCoCrFeNi High Entropy Alloys at Room and Cryogenic Temperatures. Intermetallics.

[16] Hu G.X., Cai X., Rong Y.H. (2010). Fundamentals of Materials Science.

